# Radial or Bilateral? The Molecular Basis of Floral Symmetry

**DOI:** 10.3390/genes11040395

**Published:** 2020-04-06

**Authors:** Francesca Lucibelli, Maria Carmen Valoroso, Serena Aceto

**Affiliations:** Department of Biology, University of Naples Federico II, 80126 Napoli, Italy; francesca.lucibelli@unina.it (F.L.); mariacarmen.valoroso@unina.it (M.C.V.)

**Keywords:** DIVARICATA, DRIF, RADIALIS, MYB transcription factors, flower symmetry

## Abstract

In the plant kingdom, the flower is one of the most relevant evolutionary novelties. Floral symmetry has evolved multiple times from the ancestral condition of radial to bilateral symmetry. During evolution, several transcription factors have been recruited by the different developmental pathways in relation to the increase of plant complexity. The MYB proteins are among the most ancient plant transcription factor families and are implicated in different metabolic and developmental processes. In the model plant *Antirrhinum majus*, three MYB transcription factors (DIVARICATA, DRIF, and RADIALIS) have a pivotal function in the establishment of floral dorsoventral asymmetry. Here, we present an updated report of the role of the DIV, DRIF, and RAD transcription factors in both eudicots and monocots, pointing out their functional changes during plant evolution. In addition, we discuss the molecular models of the establishment of flower symmetry in different flowering plants.

## 1. Introduction

**Figure 1:**
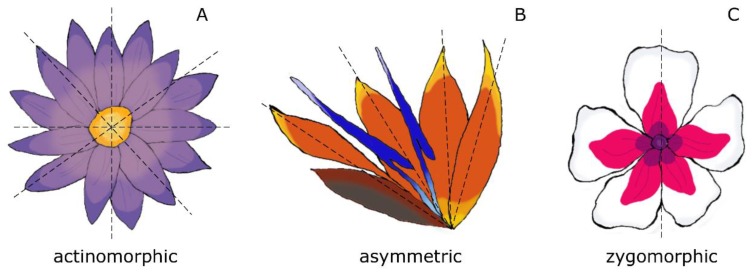
Different types of floral symmetry; (**A**) radially symmetric flower (actinomorphic), (**B**) asymmetrical flower, (**C**) bilaterally symmetric flower (zygomorphic).

The success of flowering plants is strictly related to the evolutionary innovations enclosed in the flower, whose ancestral form can be dated back ≈ 140–250 million years ago (Mya) [1,2,3,4]. The extraordinary morphological diversity displayed by flowers, affects the shape, size, and color of the perianth, as well as the disposition of the floral organs, resulting in different symmetry types, among which radial symmetry (actinomorphy) represents the ancestral state [4,5]. During the first angiosperm radiation (late Cretaceous, ≈ 93–89 Mya), the first changes from radially symmetric to asymmetric flower appeared [6,7]. The asymmetric flower is considered plesiomorphic to the bilaterally symmetric flower (zygomorphic), which evolved later during flower evolution (Paleogene, ≈ 65–34 Mya) (Figure 1) [6]. However, the transition from radial to bilateral symmetry has occurred independently many times during flower evolution [8], possibly as a result of the adoption of different pollination strategies. In fact, radial symmetry allows pollination by many types of insects, while bilateral symmetry tends to promote interactions only with specific pollinators that have often coevolved with the bilaterally symmetric flowers; for example, the bilateral flowers of the orchid genus *Ophrys* are visited by male wasps attracted by the high resemblance of the lip to female insects [9,10,11].

**Figure 2:**
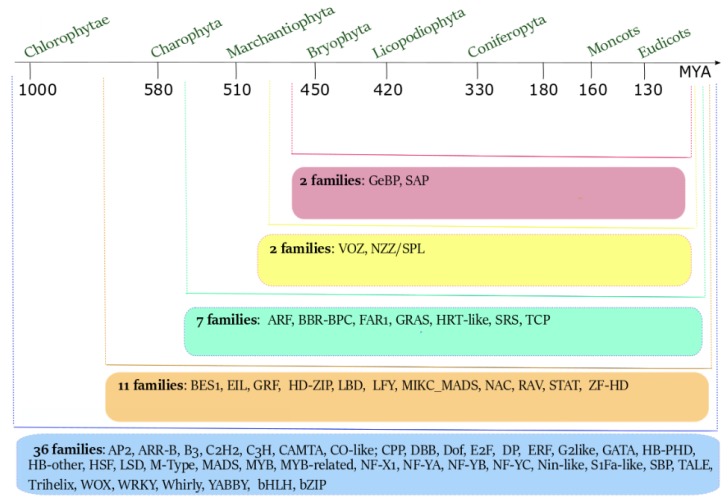
The evolution of the number of transcription factors (TF) families from Chlorophytae to Eudicots. Starting from Chlorophytae, the number of TF families increased in number and type. The number of ancestral TF families present in Chlorophytae was 36. New TF families have originated in Charophyta (11) and in Marchantiophyta (7). The last appearance of new TF families occurred before the origin of Bryophyta, with the recruitment of 4 new families (adapted from [12]).

The molecular basis of floral symmetry, such as that of other relevant processes regarding the development, life cycle, and metabolism of plants, is regulated by the action of specific transcription factors (TFs). During evolution, the number of plant TF families has expanded, ranging from 36 in Chlorophyta to 58 in Eudicots (Figure 2) [12]. The progressive increment of the number of TF families is probably linked to the increase of plant and flower complexity, to the number of genome duplications [12] and, more generally, to the evolution of plant genome complexity [13].

The classification of the TFs in different families is based on the presence of specific domains able to bind to a regulatory target sequence. Some TF families are very ancient, are present in all the plant lineages, and have assumed different roles during evolution. Among them, the MADS-box family is one of the most ancient, present since the evolution of the Chlorophyta [12]. The MADS-box TFs are involved in a wide range of developmental pathways, from spore germination [14], gametophyte and sporophyte generation [14,15], the formation of motile flagella in sperms of non-seed plants [16], to root development [17,18,19], abiotic stress responses [20], tuber dormancy [21], and fruit expansion [22]. In addition, they have been recruited in the pathway that drives the determination of flower organs, as explained by the canonical ABCDE model (mainly in eudicots) and its modifications (e.g., the fading borders in basal angiosperms, magnoliids, and basal eudicots, and the orchid code model in orchids) [23,24,25,26,27,28].

## 2. The MYB Transcription Factors

The MYB TF family, together with the MADS-box TFs, was present during plant evolution, starting from the Chlorophyta lineage (Figure 2) [12]. The MYB TFs have been initially identified in species distantly related to the plant kingdom (e.g., human, chicken, mouse, and fruit fly) as involved in the oncogenic process [29,30,31,32,33,34]. The first MYB TF isolated in plants was COLORED1 from *Zea mays*, which was involved in anthocyanin synthesis [35], and its homolog has been found in *Arabidopsis thaliana* [36]. Subsequently, MYB TFs have been identified in all eukaryotic organisms, revealing a protein structure conserved during evolution. All the MYB proteins are characterized by the presence of a variable number (from one to four and more) of MYB repeats (R). The R sequence is composed of ~ 52 amino acids and includes three regularly spaced residues of tryptophan or other aliphatic amino acids [37]; this structure forms a hydrophobic core composed of α-helices, where two helices adopt a helix–turn–helix (HTH) conformation [37,38]. This structure is necessary for DNA binding and protein–protein interactions [38,39].

Based on the number of R repeats, the MYB TFs are classified into 4R, 3R, 2R, and 1R-MYB types.

The 3R-MYBs have three R repeats (R1R2R3) and originated before the divergence between animals and plants [40,41,42]. They are mainly involved in the regulation of the cell cycle both in animals (as in humans, zebrafish, and fruit fly) [43,44,45] and plants (as in *A. thaliana*) [46].

The 2R-MYBs are plant-specific, have two R repeats (R2R3), and are the largest group of plant MYB TFs [47,48,49,50]. They are involved in various plant-specific processes such as the response to hormones, the identity of specific cell types, and regulation of secondary metabolism [51].

**Figure 3:**
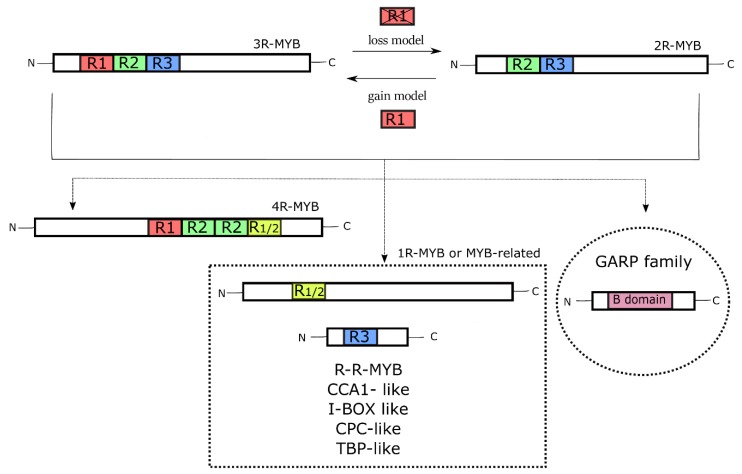
The evolution of the different types of MYB TFs. The alternative ‘gain’ or ‘loss’ model explains the origin of the 3R- and 2R-MYB types through the acquisition or deletion of the R1 repeat, respectively. The 4R-MYBs (with four R repeats) and the 1R-MYBs (or MYB-related ones) have evolved from these two types, with the last divided into five classes, all with a single R repeat. The GARP family of TFs is evolutionarily distant from the MYBs, even though its B-domain assumes a three-dimensional structure similar to that of the MYB domain.

Two hypotheses have been proposed on the evolution of the 2R- and 3R-MYB types, known as ‘the gain model’ and ‘the loss model’ (Figure 3). The gain model hypothesizes the existence of an ancient 2R-MYB before the animal-plant divergence. A subsequent intragenic domain duplication resulted in the origin of the 3R-MYB type, with the gain of another R repeat (R1), followed by the lineage-specific extinction of the 2R-MYB type in animals [47,48]. In contrast, the loss model considers the 3R-MYB type the ancestor of the whole MYB superfamily. After the divergence between animals and plants, the lineage-specific loss of the R1 repeat in duplicated 3R-MYBs gave rise to the 2R-MYB type in plants [42,47,49,50].

The 4R-MYB type includes proteins containing two duplicated repeats (R1R2-R2R1/2), and little is known about their function in plants [47]. Other MYBs, the ‘so called’ MYB-related TFs (1R-MYB type), are proteins with a variable number of R repeats and an atypical MYB domain. A large number of 1R-MYB proteins contain a single R repeat (R1/2 or R3) [47]. Based on their motifs, the 1R-MYBs are divided into five different sub-groups: R-R-MYB, CCA1-like, I-box-binding-like, CPC-like, and TBP-like [52,53]. They are involved in different basal processes, for example, regulation of the circadian clock and epidermal cell differentiation in *A. thaliana,* and binding telomeric DNA regions in *Petroselinum crispum* and *Z. mays* [54].

Some TFs, for example, the GARP proteins, are related to the MYB superfamily, although evolutionarily distant [51]. These proteins are characterized by the presence of the B-domain, whose three-dimensional structure is similar to the MYB repeat: a three-helix structure containing a variant of the HTH motif [55]. The GARP proteins are involved in different processes, such as differentiation of the photosynthetic leaf cell type (GOLDEN2 from *Z. mays*), regulation of phosphorus metabolism (PSR1 from *Chlamydomonas reinhardtii*), and control of organ polarity (KANADI from *A. thaliana*) [51,55].

Some plant MYBs have an important function in flower development. In particular, several studies have dissected the role of three MYB TFs, DIVARICATA (DIV), RADIALIS (RAD), and DIV-and-RAD-interacting-factors (DRIF), during the establishment of dorsoventral asymmetry of the flower in the snapdragon *A. majus* [56,57]. Studies in other species have then revealed that these proteins are also involved in many other processes. In the following sections, we review the most recent studies regarding these three MYB TFs, focusing our attention on their structure, evolution, functions, and interactions.

### 2.1. Structure and Evolution of DIV, DRIF, and RAD

The DIV TF belongs to the CCA1/R-R-MYB type [58,59]. In most species, the protein is ~276 amino acids long and has two MYB domains, MYBI, and II. At the N-terminus, the MYBI domain is composed of approximately 44 amino acids. It is an atypical MYB domain, where the last of the three regularly spaced tryptophan residues is replaced by a tyrosine (-**W**-**X**_23_-**W**-**X**_20_-**Y**-). It is also called the R-R(A) domain and is closely related to the I-box-binding domain proteins [52]. The MYBII domain, or R-R(B) domain, is located at the C-terminus. It is ~ 51 amino acids long and is closely related to the CCA1 domain proteins [52]. It is also known as the SHAQKYF-type MYB domain due to the presence between the second tryptophan and the last tyrosine of the SHAQKYF amino acid sequence. A small group of MYBs called DIV-like (DIVL) is strictly related to DIV, even if showing a single MYB domain [57]. The DIVL proteins are characterized by the replacement of the MYBI domain with a short, conserved motif called R-motif (R/KLFGV), whereas, at the C-terminus, they present the canonical MYBII domain [57].

The DIV and DIVL proteins evolved from an ancestral MYB protein present in the red algae and contain only the SHAQKYF domain [57]. After the divergence of red and green algae, the duplication of the SHAQKYF domain resulted in the origin of the MYBI and R motifs [57].

The DRIF proteins (~ 255 amino acids long) belong to the R-MYB type and contain two conserved domains. The first domain is an atypical MYB domain, composed of 46 amino acids. Differently from the MYB domain of DIV, in the DRIF MYB domain, tyrosine replaces the central tryptophan of the canonical MYB domain (-**W**-X_23_-**Y**-X_20_-**W**-). The second conserved domain is DUF3755, whose function is still unknown, which is present in all the DRIF proteins and composed of 66 amino acids [56].

Similarly to the DIV proteins, the evolutionary origin of the DRIF proteins and the appearance of the DUF3755 domain occurred after the divergence between red and green algae [57]. The high similarity between the DIV MYBI and the DRIF MYB domain suggests that their origin is from a common ancestor. A possible hypothesis to explain the origin of the DRIF genes assumes that, after whole duplications of the *DIV* gene, a copy of the ancestral *DIV* gene lost the region encoding the SHAQKYF domain, giving rise to the *DRIF* gene ancestor. Alternatively, a gene fusion event may have driven the union of the region encoding the MYBI domain and that encoding the DUF3755 domain, present in a different locus; the latter hypothesis is supported by the absence of the MYBII domain in the DRIF proteins, even in basal species, and to the presence of both the MYBI and DUF3755 domains, even in green algae [57].

RAD is a small interfering peptide (siPEP) belonging to the I-box-binding-MYB type [58]. RAD is composed of ~ 99 amino acids and contains a single MYBI domain. As in the DIV proteins, the MYBI domain of RAD presents the substitution of the canonical tryptophan residue with a tyrosine. Compared to DIV and DRIF, the RAD proteins evolved later during the evolution of the MYB TFs. In fact, the *RAD* genes were present, starting from gymnosperms [57]. They evolved from an ancestral, duplicated *DIV* gene that lost the region encoding the MYBII domain, possibly through deletion or alternative splicing events [57,60].

### 2.2. Roles and Interactions of DIV, DRIF, and RAD

The DIV, DRIF, and RAD TFs are involved in the processes that control different aspects of plant growth, reproduction, and metabolism. For example, in *A. thaliana,* the *AtDIV2* gene controls seed germination in an ABA-dependent manner. The loss-of-function mutants *atdiv2,* have slow seed germination and high levels of ABA, suggesting that the ABA and MYB pathways are connected and involved in seed germination [61]. In *Plantago lanceolata,* the *PlDIV* gene, orthologous of the *DIV* gene of *A. majus*, has a role in the regulation of cell proliferation during stamen development [62]. Another DIV gene, the *MID1* gene of *Oryza sativa*, is involved in anther development and drought stress response [63]. When the plant is subjected to drought stress, the higher expression of the *MID1* gene regulates some genes related to anther development (e.g., *KAR, MS*, etc.) and genes related to drought and ROS-scavenging response (e.g., *Hsp17.0, CYP707A5,* and *PODs*), improving rice yield in arid climates [63]. Finally, in the Mediterranean orchid *Orchis italica,* the DIV proteins are thought to be involved in different processes, such as reproductive organ formation and leaf development [64].

Compared to the knowledge about the functional roles of the DIV proteins in different plant species, less is known about the function of the DRIF proteins and their DUF3755 motif. Recent studies in *Populus trichocarpa* have revealed the ability of the DUF3755 domain to interact with the WUSHEL-RELATED HOMEBOX (WOX) and KNOTTED1-LIKE HOMEBOX (KNOX) proteins. This interaction seems to be involved in different processes inside the plant, such as embryogenesis, floral organ development, staminal cell regeneration, meristem identity maintenance, and lignification [65].

Like DIVs and DRIFs, the RAD proteins also play diverse roles in the different plant species. For example, in *Solanum lycopersicum* Lefsm1, a SANT/MYB1-like1 protein seems to be involved in the early stage of fruit and plant development [66]. In *A. thaliana*, another SANT/MYB1 protein (RSM1) is involved in the apical hook formation [67], acts as a positive regulator of the sensitivity of ABA signaling during seed germination, and regulates the salinity levels [68]. These functions are also conserved in monocot species, such as *O. sativa* [69]. Furthermore, both in dicots (e.g., *Senecio vulgaris, Gossypium barbadense*) and monocots (e.g., *O. italica*) RAD proteins are involved in leaf formation [64,70,71].

During evolution, the DIV, DRIF, and RAD proteins have acquired the capability to interact with each other, forming a regulatory block known as the DDR module [57]. This ability has evolved since the emergence of the DIV and DRIF proteins in green algae. These two proteins can physically interact, forming the regulative heterodimer DIV/DRIF. Subsequently, when the RAD protein appeared in gymnosperms, the formation of a new regulatory heterodimer (RAD/DRIF) has been possible as a consequence of the ability of RAD to interact with DRIF. The presence of the MYBI domain in the DIV, DRIF, and RAD proteins suggests that this domain might be involved in the protein–protein interaction at the bases of the DDR module [57], whereas the MYBII domain of DIV is involved in DNA binding [56,72]; it therefore appears that the DDR module evolved in gymnosperms and persisted in flowering plants. Its origin seems to be related to the establishment of new regulatory networks in relation to the increasing plant complexity [57,73].

The functional role of the DDR module has been studied in different species. For example, in *S. lycopersicum,* three MYB TFs, SlMYBI (an R-RMYB type similar to DIV), SlSFM (a SAINT-MYB-like1 similar to RAD), and SlSFB (containing a DUF3755 domain) can interact following the DDR scheme and regulate cell expansion during tomato fruit development. Specifically, the heterodimer SlSFM/SlSFB suppresses cell expansion, inhibiting the positive regulatory function of SlMYBI. On the contrary, the heterodimer SlMYB/SlSFB induces the activation of genes involved in cell elongation [74]. In addition, the DDR module regulates crucial points during the establishment of flower symmetry.

## 3. The Molecular Basis of Flower Symmetry

**Figure 4:**
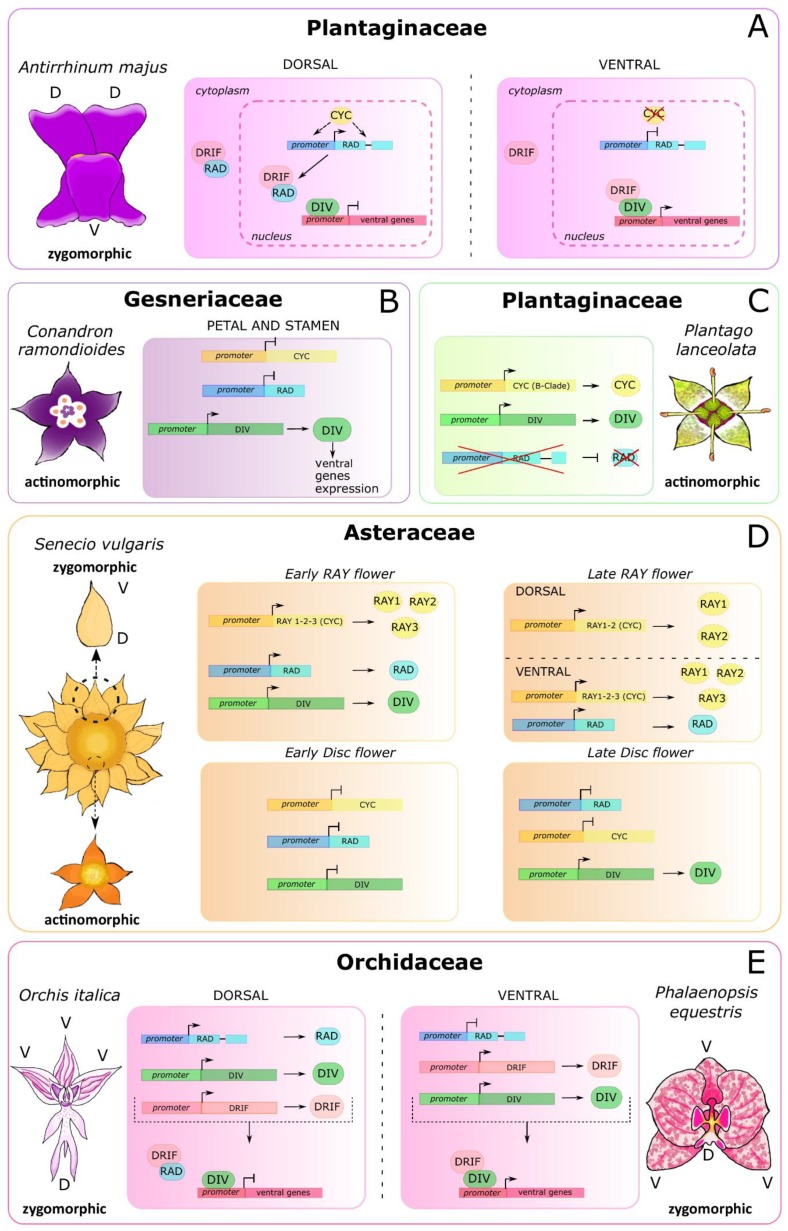
Molecular models explaining the establishment of floral symmetry in different plant families. The key factors belong to the TCP (CYC) and MYB (DIV, DRIF, RAD) TF families. The molecular pathways established in different species vary in relation to the symmetry type of the flower. Within the Plantaginaceae family, two examples illustrate the regulative network when the flower has dorsoventral asymmetry (**A**, *A. majus*) or radial symmetry (**C**, *P. lanceolata*). **B**) The molecular pathway that drives the radial symmetry of the *Conandron ramondioides* flower (Gesneriaceae). **D**) The complex symmetry of the ray and disc florets within the capitulum of *Senecio vulgaris* (Asteraceae). **E**) The bilateral flower of orchids (Orchidaceae). As a consequence of the resupination, the model is rotated of 180° with respect to that of *A. majus*.

The genetic control of floral symmetry was first dissected in the snapdragon *A. majus*, whose flower has a dorsal and a ventral part with distinct characteristics (dorsoventral asymmetry) [75]. The *Antirrhinum* flower has five petals in three different relative positions: two dorsal, two lateral, and one ventral. In addition, the dorsal stamen is aborted, and the abaxial stamens are longer than the lateral ones [76]. The ventral identity of the snapdragon flower is established through the interaction between the two MYB TFs DIV and DRIF. In the ventral region, the DRIF protein forms a heterodimer with the protein DIV. This complex migrates from the cytoplasm to the nucleus, where it activates the transcription of downstream ventralization genes, still unknown. The presence of the DIV target sequence (5′-GATAA-3′) in the *DIV* promoter suggests that the DIV/DRIF complex could autoregulate the transcriptional activity of *DIV* itself [57,77]. The DIV and DRIF proteins are also present in the dorsal region of the *Antirrhinum* flower; however, their interaction is inhibited due to the presence of the siPEP RAD [60,78,79]. In fact, in the dorsal region, RAD binds DRIF, thus preventing its interaction with the DIV protein. In this way, although presen in the dorsal part of the flower, the DIV protein is not able to activate the ventralization genes [56]. The expression of the *RAD* gene in the dorsal region of the snapdragon flower is regulated by CYC and DICH, two paralog TCP TFs belonging to the CYC2 clade and expressed only in the dorsal part of the *Antirrhinum* flower [76,80,81]. The transcriptional regulation of *RAD* is accomplished through the binding of the CYC protein to its target sequences (5′-GGNCCC-3′), located within the *RAD* promoter and intron [82] (Figure 4A).

The study of the molecular mechanisms underpinning floral symmetry is useful in order to understand the evolution of the different species of angiosperms. A conserved expression pattern of the *CYC* genes in the dorsal floral organs of bilaterally symmetric flowers has been demonstrated in a number of species belonging to Fabales, Brassicales, Asterales, and Malpighiales [83,84,85,86]. Bilateral symmetry of the flower is predominant in plant orders with a large number of species, such as the Lamiales (that include *A. majus*), confirming the hypothesis that bilateral flower symmetry could be positively related to speciation rates [87].

Within Gesneriaceae (Lamiales), the flowers generally have bilateral symmetry [5]; however, during the evolution and diversification of this family, there have been many independent reversion events from bilateral to radial symmetry [88]. These transitions of flower symmetry are associated both to mutations of specific genes and/or to alterations of their expression profile [89,90,91]. For example, *C. ramondioides* have radially symmetric flowers. In this species, there is a change of the expression pattern of the homolog genes involved in the floral symmetry of *A. majus*. The loss of the expression of the *CrCYC* and *CrRAD* genes in petals and stamens, and the ubiquitous expression of the *CrDIV* gene, are associated to the ventralization of the flower. In addition, the co-expression of *CrDIV* and *CrRAD* in gynoecium and stamens at the same developmental stage suggest the loss of their antagonistic role in this species [92] (Figure 4B).

The tribe Veroniceae is another group of Lamiales where the transition of symmetry from bilateral to radial is observed. Radial symmetry of the flower in *Plantago* is associated with the wind-pollination syndrome and depends on the alteration of the floral symmetry network that involves the homologs of the *CYC*, *DIV,* and *RAD* genes of *A. majus*. In fact, in *Plantago,* there was a loss of the *CYC* A-clade genes. At the same time, there was the expansion of the expression domain and the possible neofunctionalization of the *CYC* B-clade genes [93]. *P. lanceolata* has a single *CYC-like* gene (the B-clade *PlCYC*) that shows a stage-specific expression profile during flower development. At the early stage, *PlCYC* is expressed in the ground tissue and in the tissue from which the pedicel derives. At the later stage, *PlCYC* is expressed in the connective tissue of the anthers, in all four stamens, and in the upper part of the filament. The absence of expression of *PlCYC* in petals might be related to the reversion to radial symmetry [94]. In addition, in *P. lanceolata,* a *DIV* ortholog (*PlDIV*) was detected, whereas *RAD* seems to be absent. The *PlDIV* gene is ubiquitously expressed in the early flower organs, as in *A. majus*; however, at a later stage, *PlDIV* is expressed in the lateral side of petals in the stamens and the ovary. The absence of the A-clade *CYCs* and the *RAD* genes in *P. lanceolata* has probably caused the loss of the network that controls the dorsal identity of the flower, while the presence and the expression profile of *PlDIV* are consistent with the total ventralization of the flower [62] (Figure 4C).

In other species, the genetic basis of floral symmetry can be still more complex, and an example is found within Asteraceae, one of the most species-rich families of flowering plants. The species that belong to this family have a peculiar inflorescence called the capitulum, which is generally composed of many compacted florets whose morphology is variable within the same capitulum. In many species, including *Senecio vulgaris*, the capitulum contains two types of flowers; the central disc florets with radial symmetry and the marginal ray florets, with bilateral symmetry [95]. In this species, the regulatory network composed of the *CYC*, *RAD,* and *DIV* genes is completely reinvented to generate the elaborate structure of the capitulum. During the early stages of the capitulum development of *S. vulgaris* (from stage 1 to 4), the CYC-like genes *RAY1-3*, and the MYB genes *SvDIV1B* and *SvRAD* are expressed only in the ray florets. Later, *RAY1* and *RAY2* are expressed all over in the ray florets, while the expression of *RAY3* and *SvRAD* is localized only in the ventral region, promoting dorsoventral asymmetry through the elongation of the ventral part of the petal. This expression pattern is in contrast with the model described in *A. majus,* in which the *CYC* gene is expressed in the dorsal part of the flower. Starting from stage 5, *SvDIV1B* is expressed both in ray florets and disc florets, even if, at stage 8, in the ray florets its expression decreases during the elongation of the ventral part of the petal, thus contributing to the establishment of dorsoventral asymmetry [70] (Figure 4D).

Another example of different development between the ray and disc florets, regulated by the specific expression profile of the *CYC* genes, is represented by *Helianthus annuus,* the common sunflower. In this species, *HaCYCc* is the ortholog of *RAY3,* and it is expressed mainly in ray florets throughout all developmental stages [96]. Notably, mutant ray florets with loss-of-function of *HaCYCc,* which have a radialized phenotype, showing the involvement of this gene in the formation of dorsoventral asymmetry of the ray florets.

In monocots, little is known about the molecular determination of floral symmetry; however, as the DDR regulatory model was established before the diversification between monocots and dicots, it is possible that it is also involved in the control of floral symmetry in monocots [59]. This hypothesis is supported by studies conducted in the Orchidaceae. In this family, the expression domains of the *DIV*, *RAD,* and *DRIF* genes are generally conserved with respect to *A. majus*. Compared to snapdragon, the orchid DDR module works with a 180° rotation due to resupination, a turn of the pedicel that occurs before anthesis in some orchid species shifting the lip, a dorsal structure, to a ventral position [97]. In the bilaterally symmetric flower of the orchids *O. italica* and *Phalaenopsis equestris*, the *DIV* and *DRIF* genes are expressed in all tissues of the perianth and can interact with each other in the lateral inner tepals, ventral structures that after resupination, take a dorsal position. In the lip of both species, the high level of RAD prevents the interaction between the DIV and DRIF proteins. RAD promotes the identity of the lip competing with DIV for the interaction with DRIF, avoiding the formation of the DIV/DRIF complex, as in *A. majus*. This model of interaction is confirmed by the expression analysis of the *DIV*, *RAD,* and *DRIF* genes in the peloric orchid mutant *Phalaenopsis* Joy Fairy Tale, which has radial flowers with three lips in the second floral whorl. In this peloric orchid, the expression level of the *RAD* gene is comparable in the lip and in the lip-like organs that replace the lateral inner tepals. Consequently, the interaction between DIV and DRIF is suppressed even in the lip-like organs, resulting in a radially symmetric flower [73] (Figure 4E).

## 4. Conclusions

In recent years, advances have been made in understanding the role of the MYB TFs in the molecular network regulating flower symmetry. During evolution, the MYB proteins DIV, DRIF, and RAD have assumed new functions, and the DDR regulatory module has been recruited for the establishment of flower symmetry. In addition to MYBs, other TF families (e.g., the TCPs through CYC) are involved in the molecular program that permits the establishment of radial or bilateral symmetry. Given the complexity of the molecular developmental networks, it is reasonable to hypothesize the engagement of further TF families. For example, the ancient MADS-box TFs family is known to play a crucial role in the development of flower organs with functions, expression domains, and interactions that have changed during evolution in relation to the different flower morphologies [23,24,25,26,27,28]. Some evidence already exists of a link between MADS, MYB, and TCP TFs. For example, in *A. majus*, the heterodimer DIV/DRIF induces the activation of the gene *AmMYBML1*, which regulates the development of ventral-specific cell type together with the MADS-box genes belonging to the B-class [98]. In addition, the CYC transcription factor is regulated by B- and C-class MADS-box genes [99]. Another promising group of TFs that are candidates to be involved in flower symmetry is the NAC family, known to be involved in the formation of lateral organ boundaries and that seem to have acquired new functions in the growth of the lip [100].

Although significant efforts have been made and some specific aspects have been clarified, the genetic basis of flower symmetry is still far from being fully understood. Future studies should try to define the connections that exist among the different TF families, in addition to the roles of the specific genes, to obtain an integrated picture of this fascinating and complex developmental process.
